# A Novel Risk Score Model Based on Eleven Extracellular Matrix-Related Genes for Predicting Overall Survival of Glioma Patients

**DOI:** 10.1155/2022/4966820

**Published:** 2022-04-29

**Authors:** Xiaodong Li, Yichang Wang, Wei Wu, Jianyang Xiang, Lei Qi, Ning Wang, Maode Wang, Hai Yu

**Affiliations:** ^1^Department of Neurosurgery, The First Affiliated Hospital of Xi'an Jiaotong University, Xi'an, Shaanxi, China; ^2^Center of Brain Science, The First Affiliated Hospital of Xi'an Jiaotong University, Xi'an, Shaanxi, China

## Abstract

Gliomas are the most common lethal primary brain tumors with variable survival outcomes for patients. The extracellular matrix (ECM) is linked with clinical prognosis of glioma patients, but it is not commonly used as a clinical indicator. Herein, we investigated changes in ECM-related genes (ECMRGs) via analyzing the transcriptional data of 938 gliomas from TCGA and CGGA datasets. Based on least absolute shrinkage and selection operator (LASSO) Cox regression analysis, a 11-ECMRG signature that is strongly linked with overall survival (OS) in glioma patients was identified. This signature was characterized by high-risk and low-risk score patterns. We found that the patients in the high-risk group are significantly linked with malignant molecular features and worse outcomes. Univariate and multivariate Cox regression analyses suggested that the signature is an independent indicator for glioma prognosis. The prediction accuracy of the signature was verified through time-dependent receiver operating characteristic (ROC) curves and calibration plots. Further bioinformatics analyses implied that the ECMRG signature is strongly associated with the activation of multiple oncogenic and metabolic pathways and immunosuppressive tumor microenvironment in gliomas. In addition, we confirmed that the high-risk score is an indicator for a therapy-resistant phenotype. In addition to bioinformatics analyses, we functionally verified the oncogenic role of bone morphogenetic protein 1 (BMP1) in gliomas *in vitro*.

## 1. Introduction

Gliomas are the most common and lethal primary tumors in adults, accounting for more than 80% of malignant primary brain tumors [1]. They have a highly infiltrative nature, strong angiogenesis, high heterogeneity, therapeutic resistance, and a rapid relapse [2–4]. Even with standard treatments, including maximum surgical resection, irradiation therapy, and chemotherapy, the prognosis for glioma patients has been barely improved [5]. Previous studies showed that the 5-year overall survival (OS) for glioma patients is less than 20% [1], and patients with glioblastoma (GBM) have a median survival time of 14.6 months [6].

Recent studies divided GBM into proneural, neural, classical, and mesenchymal types based on their transcriptional profiles [7]. It has also been shown that the mesenchymal subtype is an aggressive subtype that is strongly linked with therapeutic resistance [8]. However, the predictive factors for gliomas are still insufficient in evaluating patients' clinical outcome since glioma patients with the same signature often have distinct clinical features [9]. Therefore, more studies are warranted to identify comprehensive predictive models.

The extracellular matrix (ECM) is the noncellular component of tissues and organs, responsible for tissue homeostasis, remodeling, and regeneration [10]. Accumulating evidence showed that the ECM is involved in malignant progression of multiple cancers, including breast cancer [11], urothelial bladder cancer [12], and liver cancer [13]. Interestingly, a study reported that glioma patients with a stiff, tenascin-rich ECM, have a mesenchymal-like phenotype and poor survival [14]. Surprisingly, a study suggested that elevated ECM stiffness can independently contribute to the aggressiveness and recurrence of GBM and predict a worse outcome of glioma patients via bypassing the isocitrate dehydrogenase 1 (IDH1) mutational protection [15]. In addition, a study found that the ECM remodeling is tightly correlated with various metabolic pathways such as glycolysis, which provides sufficient energy and biosynthetic substrates during tumorigenesis [16]. Moreover, a pan-cancer analysis found that upregulated ECM genes are tightly related to the transformation from immunoactive M1 to immunosuppressive M2 macrophages [17]. A recent study revealed that ECM modifications can enhance the therapeutic effect of immunotherapy on GBM [18]. Thus, the ECM is a potent candidate for outcome prediction in glioma patients. However, studies focusing on the ECM characteristics in gliomas are still lacking.

In this study, we focused on the expression profile of ECM-related genes (ECMRGs) from The Cancer Genome Atlas (TCGA) and the Chinese Glioma Genome Atlas (CGGA) datasets. The results suggested that glioma patients can be classified into 2 clusters with distinct molecular features and clinical outcomes. In addition, an ECMRG signature was constructed to predict the OS of glioma patients. Univariate and multivariate Cox regression analyses showed that the risk signature is an independent prognostic model. The receiver operating characteristic (ROC) curves and calibration curves indicated that this risk signature is a good predictor of OS. Moreover, bioinformatics analyses indicated that the risk signature is closely related to oncogenic pathways in gliomas and that the signature ECMRGs have significant impacts on the regulation of metabolic status and tumor immune microenvironment (TIME). Additionally, we found that the high-risk score is indicative of a therapy-resistant phenotype. Lastly, we confirmed that bone morphogenetic protein 1 (BMP1), which is part of the risk signature, is strongly linked with malignant characteristics of gliomas.

## 2. Materials and Methods

### 2.1. Included Patients and Datasets

A total of 938 glioma samples have been investigated in this study. mRNA expression data of two public datasets were obtained, including TCGA RNA sequencing (RNA-seq) dataset [19] and CGGA RNA-seq dataset [20]. For TCGA RNA-seq data (629 samples), level 3 mRNA expression profiles integrated by the Illumina HiSeq RNASeqV2 system were derived from TCGA project (https://xenabrowser.net/datapages/). The normalized count reads from the preprocessed data (sequence alignment and transcript abundance estimation) were log2 transformed. For the CGGA RNA-seq data (309 samples), the detailed pipeline has been reported before (http://www.cgga.org.cn). The clean reads were aligned to human genome reference (hg19), and sequencing read counts for each RefSeq gene were calculated using RSEM. The normalized expression levels of different samples were log2 transformed and used in this study.

TCGA dataset was used as the discovery dataset. The corresponding clinical data of 629 glioma samples were collected from TCGA dataset (https://portal.gdc.cancer.gov/). Similarly, the CGGA dataset was included as the external validation dataset. The corresponding clinical information of 309 glioma samples was obtained from the CGGA website (http://www.cgga.org.cn). We have summarized the clinicopathological characteristics for all patients in (Table 1 and Table S1). This study was approved by the Institutional Review Boards (IRB) of the First Affiliated Hospital of Xi'an Jiaotong University (XJTU).

### 2.2. Data Processing and Risk Score Construction

**Figure 1:**
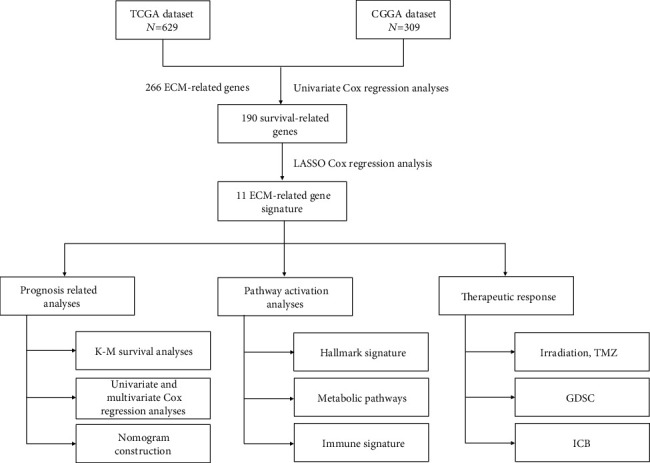
The flow chart showing the process of the study design.

We performed a comprehensive analysis with TCGA and CGGA datasets to identify and construct a clinically translatable gene signature that captured ECM alternations of tumor cells, as shown in Figure 1, hereafter referred to as the ECMRG signature.

Patients with complete survival information were analyzed in this process. To obtain the ECMRG signature, the ECMRGs were used as criteria for screening (https://maayanlab.cloud/Harmonizome/gene_set/Extracellular+matrix+organization/Reactome+Pathways). In TCGA and CGGA datasets, we analyzed the survival prediction value of 266 ECM genes via univariate Cox regression analysis. In total, 190 ECMRGs were found to be strongly associated with a patient's prognosis in both datasets.

Next, the 190 ECMRGs were subsequently analyzed using LASSO regression [21], to select the most powerful prognostic biomarkers. Using the “glmnet” package (4.0-2) in R, the LASSO regression model was selected to minimize the overfitting and identify the most significant survival-associated ECMRGs in gliomas (10-fold cross-validation). Of the 190 ECMRGs, 11 ECMRGs were identified and selected. A formula that combined the relative expression of the 11 ECMRGs and their respective coefficients was constructed. ECMRG signature = *β*1Exp1 + *β*2Exp2 + ⋯+*β*11Exp11 (*βi* and Exp*i* represent the regression coefficient and the gene expression level, respectively) (Table S2). Based on the above formula, we calculated the risk score (RS) for each sample in TCGA dataset, and the median value was manually chosen as the threshold for high and low. Similarly, the risk score was obtained from the CGGA dataset using same formula. The predictive accuracy was analyzed using a time-dependent ROC curve [22].

### 2.3. Consensus Clustering

Most variable genes were identified by median absolute deviation (MAD) and used for consensus clustering. The R package “ConsensusClusterPlus” (1.52.0) was used in R (4.0.0) for consensus clustering analysis and graphic generation.

### 2.4. Nomogram Construction

R package “rms” (6.0-1) was used to establish the prediction model, incorporating the risk score and clinicopathologic characteristics (age, grade, and status of 1p19q codeletion). The Schoenfeld residual test was performed to test the proportional hazards (PH) assumption [23] for all variables included in the nomogram model by using the survival (3.2-13) and survminer package (0.4.8), and age was found to be nonproportional. Subsequent models were stratified by age into young (≤47 years) and old (>47 years) to satisfy the PH hypothesis [24]. The calibration of the nomogram was assessed using calibration curves. Harrell's C-index was calculated to assess the discrimination.

### 2.5. Pathway Activation Analyses

For principal component analysis (PCA), R package “princomp” was used to explore the difference within the high-risk and low-risk groups.

Gene set variation analysis (GSVA) is a nonparametric and unsupervised gene set enrichment method that can estimate the score of a certain pathway or signature based on transcriptomic data [25]. Thus, we firstly achieved the hallmark gene sets (http://www.gsea-msigdb.org/) and 114 metabolism-relevant gene signatures [26] from previous studies. By using “GSVA” package (1.36.2), each sample received 164 scores corresponding to 50 hallmark pathways and 114 metabolism signatures. The results were visualized via the “pheatmap” package (1.0.12).

Gene set enrichment analysis (GSEA) was carried out using a well-known online tool (http://software.broadinstitute.org/gsea/index.jsp). Based on the median level of risk score, glioma samples in each dataset were divided into two groups. Normalized values for gene expression were used as input for GSEA software (4.0.3). *P* values were calculated by permuting the genes 1,000 times. During this process, the risk score was regarded as a phenotype. The h.all.v7.2.symbols.gmt in the Molecular Signatures Database (MSigDB) was selected as the reference gene set, and *P* adjusted value < 0.05 was chosen as the cut-off criteria.

### 2.6. Analyses of Immune Signature

We employed a previously reported method to assess the immune infiltrations in gliomas [27]. The 782 metagenes for 28 immune cell subpopulations were obtained from Charoentong et al. [28]. The immune infiltration levels were quantified using enrichment scores (metascore) calculated by single-sample gene set enrichment analysis (ssGSEA) via the “GSVA” package (1.36.2) in R. Unsupervised clustering was performed using the calculated metascores in TCGA and CGGA datasets and visualized via the “pheatmap” package (1.0.12) and “corrplot” package (0.84) [29].

### 2.7. Association Analyses of the Risk Signature and Drug Response

We obtained the transcriptional profiles of glioma cell lines from the Genomics of Drug Sensitivity in Cancer (GDSC, http://www.cancerrxgene.org/downloads). The cell lines were subgrouped into the high- and low-risk groups. Next, we analyzed the difference of the drug sensitivity to a variety of drugs available in the GDSC between these two groups. The results were visualized via heat map. Lastly, the immune-checkpoint blockade (ICB) response was evaluated by the Tumor Immune Dysfunction and Exclusion (TIDE, http://tide.dfci.harvard.edu/) algorithm using mRNA expression data of TCGA and CGGA datasets [30].

### 2.8. *In Vitro* Cell Cultures

U87 glioma cell lines were provided by the First Affiliated Hospital of Xi'an Jiaotong University. Tumor cells were cultivated in DMEM-F12 medium containing 10% vol FBS supplement and 1% penicillin-streptomycin antibiotics. The culture medium was changed every 3-4 days.

### 2.9. Lentivirus Production and Transduction

Plasmid DNA was collected by using a mini plasmid purification kit (TIANGEN). HEK293T cells were transfected with the pLKO.1-TRC cloning vectors (Addgene) and two packaging plasmids psPAX2 and pMGD2 using the Calcium Phosphate Cell Transfection Kit (Beyotime). Medium containing lentivirus was collected at 24 hours and 48 hours. PEG-8000 (Beyotime) was used to precipitate the lentivirus. U87 glioma cells were incubated with medium containing lentivirus for 14 hours in the presence of 8 *μ*g/ml polybrene. Then, change to medium described above and continue to cultivate for 72 hours. The target sequence for shRNA used in this study was shBMP1#1: CACCTCCCAGTACAACAACAT and shBMP1#2: GCGCTACTGTGGCTATGAGAA.

### 2.10. RNA Isolation and Quantitative Real-Time Polymerase Chain Reaction (qRT-PCR)

RNA was isolated by using the RNeasy mini kit (QIAGEN) according to the manufacturer's protocol. The qRT-PCR analysis was performed based on methods as previously described [31]. The primer sequences applied in the study include the following: BMP1 (forward GGGTCATCCCCTTTGTCATTG; reverse GCAAGGTCGATAGGTGAACACA) and GAPDH (forward: GGAGCGAGATCCCTCCAAAAT; reverse: GGCTGTTGTCATACTTCTCATGG).

### 2.11. Cell Viability Assay

Viability of U87 cells was determined using the AlamarBlue reagent (Thermo Scientific). Tumor cells were seeded into a 96-well plate at the density of 1000 cells per well. After the indicated period of time, each well was added with the AlamarBlue reagent, and fluorescence was measured (excitation 515-565 nm, emission 570-610 nm) after 6 hours using the Synergy HTX Multi-Mode Reader (BioTek).

### 2.12. Colony Formation Assay

Colony formation assays were carried out to detect self-renewal ability of U87 cells. Tumor cells were seeded at 1000 cells per well in a 6-well plate in the medium described above. After about 10 days of cultivation, the cells were fixed with methanol and stained with methylene blue. The number of clones was counted to assess the self-renewal ability of U87 cells.

### 2.13. Wound Healing Assay

The wound healing assays were applied to determine the migratory ability of U87 cells. Tumor cells were seeded at 1∗10^5^ cells per well in 6-well plates and were cultured in the medium. After 24 hours, a sterile pipette tip was used to produce wound lines. Images were taken using an inverted microscope after 0 and 36 hours. The leading edges were marked by black lines, and the relative distance of the borders was measured by ImageJ software.

### 2.14. Transwell Migration Assay

Transwell migration assays were performed to assess the migration ability of U87 cells. Tumor cells were seeded in the upper chamber containing serum-free medium at a density of 2∗10^4^ per well. The lower chamber was added with medium containing 10% vol FBS supplement. After 16 hours, the cells in the lower chamber were removed, and images were taken under an inverted microscope.

### 2.15. Statistics

The statistical analyses were carried out using the R software (version 4.0.0, “pheatmap” package (1.0.12) for expression heat map visualization, “survivalROC” (1.0.3) and “pROC” package (1.16.2) for ROC analysis, “clusterProfiler” package (3.16.1) for KEGG analysis, “glmnet” package (4.0-2) for LASSO analysis, “GSVA” package (1.36.2) for immune infiltration analysis, “corrplot” package (0.84) for correlation heat map visualization, and “circlize” package (0.4.10) for circle plot), SPSS (version 22.0, univariate and multivariate Cox regression analyses), and Prism 6 (GraphPad Software, K-M plot and dot plot). The Chi-squared test was carried out to explore the differences in the clinicopathologic characteristics between the 2 clusters of patients. The two-tailed *t*-test was applied to evaluate statistical significance between two groups. To evaluate the independent prognostic value of each factor, univariate and multivariate Cox regression analyses were carried out. The Kaplan-Meier (K-M) analysis was performed to investigate the glioma patients' OS. Patients were classified into two groups according to the median of each gene expression or risk score for OS analysis. Pearson's correlation coefficient was calculated in correlation analysis. *P* < 0.05 was regarded as statistically significant.

## 3. Results

### 3.1. Exploration of the ECMRG Signature in Gliomas

**Figure 2:**
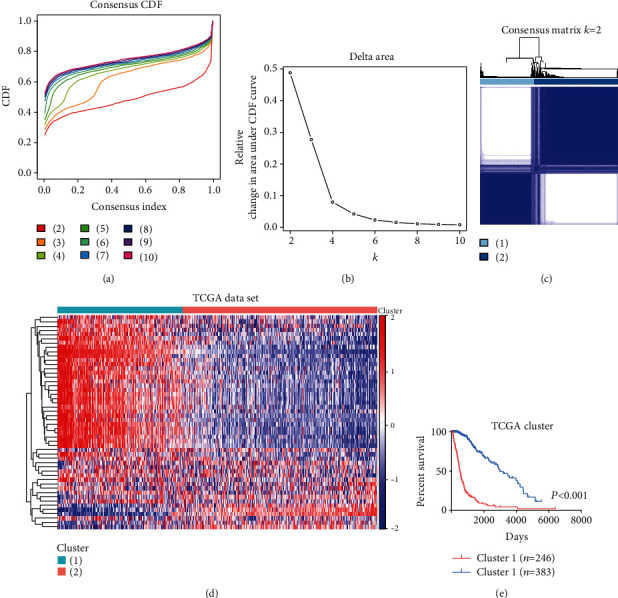
Consensus clustering for the ECMRGs in glioma patients in TCGA dataset. (a) Consensus clustering CDF for *k* = 2 to *k* = 10. (b) Relative change in area under CDF curve for *k* = 2 to *k* = 10. (c) Consensus clustering matrix of 629 samples from TCGA dataset for *k* = 2. (d) Heat map of 2 clusters constructed by the top 50 differential expression genes. (e) K-M survival analysis of patients from 2 clusters classified by consensus clustering.

To investigate the potential oncogenic roles of the ECM in patients with gliomas, we analyzed TCGA dataset using the consensus clustering method. Cumulative distribution function (CDF) and consensus matrices were constructed to determine the optimal number of subgroups. The results indicated that glioma patients can be classified into two robust clusters (Figures 2(a)–2(c)), in which distinct ECMRG expression features were represented using a heat map (Figure 2(d)). The Chi-squared test indicated that patients in cluster 1 are characterized by the malignant features, including older age (>47 years), higher grade, classical or mesenchymal subtypes, wild-type IDH, 1p/19q non-codeletion, and nonmethylation of O^6^-methylguanine-DNA methyltransferase promoter (MGMTp). Meanwhile, patients in cluster 2 demonstrated opposite clinical features (Table 1). In addition, K-M survival analysis showed that OS is shorter in cluster 1 compared with that in cluster 2 (Figure 2(e)). To confirm these findings, we performed the same analyses using the CGGA dataset and obtained similar results (Figure S1 and Table S1). These results suggest that the ECM is closely linked with the molecular features and clinical prognosis of glioma patients.

**Figure 3:**
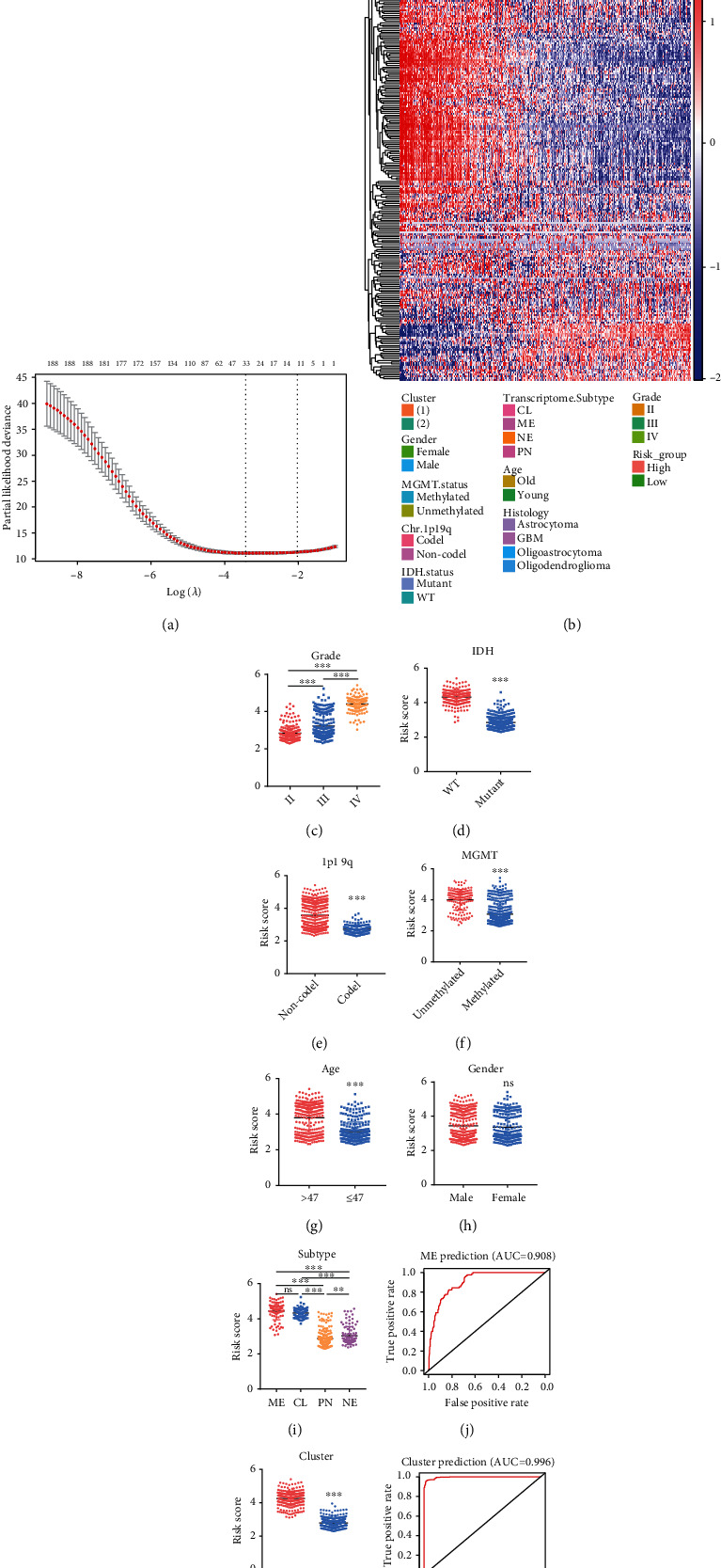
Identification of the ECMRG signature in TCGA dataset. (a) Cross-validation for tuning parameter selection in the LASSO regression model. (b) Heat map showing the expression profiles of 266 ECMRGs and corresponding clinical characteristics in TCGA dataset. (c–i) Dot plots comparing the risk score for glioma patients stratified by WHO grade, IDH mutation status, 1p/19q codeletion status, MGMTp methylation status, age, gender, or molecular subtypes of gliomas. (j) ROC curve analyzing the predictive role of the risk signature for the mesenchymal subtype. (k) Dot plot comparing the risk score for glioma patients between cluster 1 and cluster 2. (l) ROC curve analyzing the predictive role of the risk signature for cluster groups. ^∗∗^*P* < 0.01,  ^∗∗∗^*P* < 0.001; ns: not significant.

Therefore, a risk signature was constructed to evaluate the predictive role of the ECMRGs in the prognosis of glioma patients. The most powerful predictive genes with nonzero regression coefficients were identified by the LASSO Cox regression model. In total, 11 genes were selected, including BMP1, BMP2, CASP3, CD151, COL8A1, LOX, PLOD3, SDC1, SERPINH1, SPP1, and TIMP1 (Figure 3(a) and Figure S2A). Consequently, the risk score of the 11-gene signature was calculated (formula mentioned in Materials and Methods) based on the genes' relative expressions and corresponding coefficients. For further verification, the risk score was also calculated using the same signature genes and regression coefficients in the CGGA dataset. Based on the median value of risk scores, the glioma patients were subgrouped into the low-risk and high-risk groups. The correlation between the ECMRG expression and clinical features in TCGA and CGGA datasets was represented by heat maps (Figure 3(b) and Figure S2B).

### 3.2. High-Risk Score Is Tightly Associated with Malignant Clinical Features in Gliomas

The above results demonstrated a potential link between the risk signature and clinical characteristics of glioma patients. To validate our observations, we performed further analyses using TCGA and CGGA datasets. The results suggested that the risk score is positively associated with the glioma grade (Figure 3(c) and Figure S2C). In addition, the risk score was significantly elevated in patients with wild-type IDH, 1p19q non-codeletion, and unmethylated MGMTp (Figures 3(d)–3(f) and Figure S2D-F). Moreover, the risk score was higher in older patients (Figure 3(g) and Figure S2G), and no significant difference was observed between male and female (Figure 3(h) and Figure S2H). Additionally, the mesenchymal subtype, which is recognized as the aggressive type of gliomas [8], had the highest risk score (Figure 3(i)). ROC curve analysis was conducted to evaluate the predictive role of the risk signature for mesenchymal subtype. Interestingly, the area under the curve (AUC) for the risk signature in predicting the mesenchymal subtype was 0.908 in TCGA dataset, highlighting the potential oncogenic role of the risk signature ECMRGs (Figure 3(j)). Lastly, we tested if the risk signature matched the previously identified cluster groups. The results showed that cluster 1 samples have a significantly higher risk score (Figure 3(k) and Figure S2I). Accordingly, the AUC for the risk signature in predicting the cluster was 0.996 (TCGA) and 0.983 (CGGA), respectively (Figure 3(l) and Figure S2J). These findings imply that the ECM may play a crucial role in the malignant progression of gliomas.

### 3.3. Prognostic Value of the ECMRG Signature

**Figure 4:**
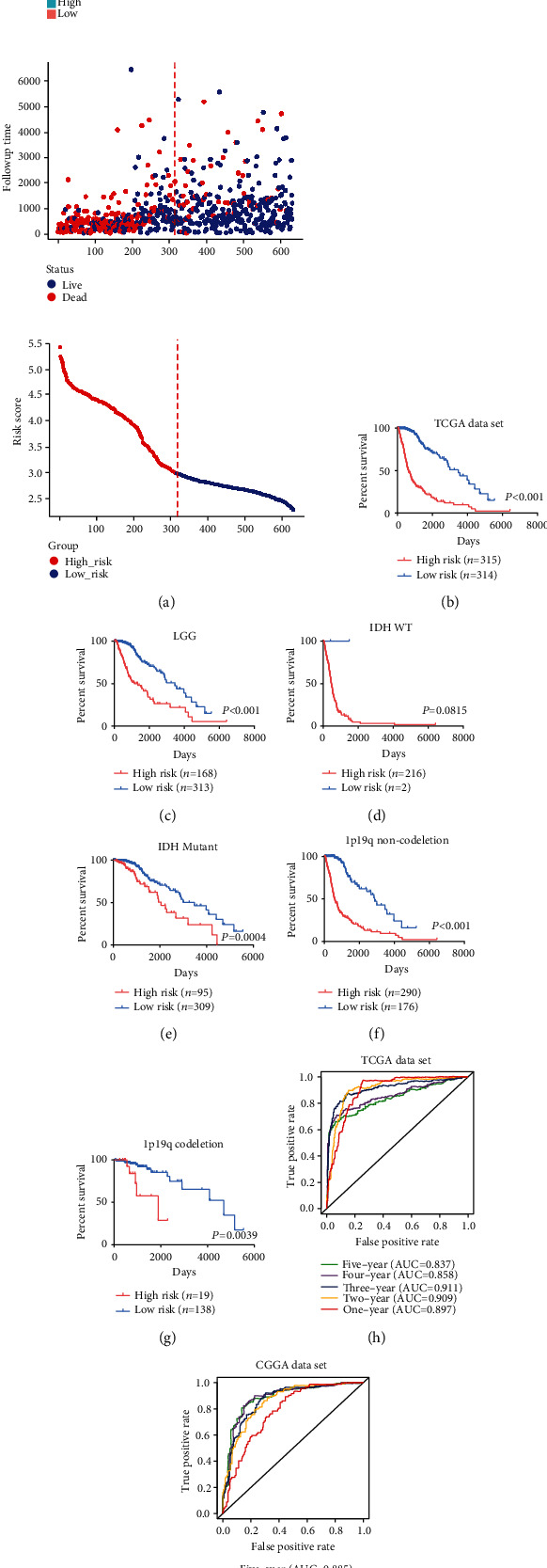
The prognostic value of the risk signature in TCGA dataset. (a) Distribution of the risk score, survival status, and mRNA expression level of 11 genes in the risk signature. (b–g) K-M survival analyses comparing OS for glioma patients in the high-risk and low-risk groups stratified by WHO grade, IDH mutation status, or 1p/19q codeletion status. (h, i) Time-dependent ROC curve analyses showing the predictive value of the risk score for 1-year, 2-year, 3-year, 4-year, and 5-year OS in TCGA and CGGA datasets.

For a more comprehensive understanding of the risk signature, the association between the mRNA expression level of each gene and patients' OS was evaluated. The results suggested that glioma patients with a high-risk score in TCGA dataset suffer worse prognosis. The patients also had significantly higher expression levels of BMP1, CASP3, CD151, COL8A1, LOX, PLOD3, SDC1, SERPINH1, SPP1, and TIMP1. Conversely, patients with a low-risk score had a better prognosis and higher expression of BMP2 (Figure 4(a)). K-M curve analyses suggested that the 11 genes can effectively distinguish the outcome of glioma patients (Figure S3). Moreover, these results were confirmed using the CGGA dataset (Figures S4 and S5A).

Next, K-M curve analyses were performed to further explore the prognostic value of risk signature in TCGA dataset. The result suggested that glioma patients in the high-risk group have worse OS (Figure 4(b)). Moreover, we classified the patients based on the histological signature and observed similar results in lower grade gliomas (LGGs) (Figure 4(c)). Because all GBM samples in TCGA dataset were assigned to the high-risk group, we could not compare their prognosis accordingly. Interestingly, we also found that there were only two patients with wild-type IDH in the low-risk group (Figure 4(d)). K-M analyses stratified glioma patients by IDH mutation, 1p/19q codeletion status, or MGMTp methylated status and showed that a high-risk score is tightly linked with worse OS (Figures 4(e)–4(g) and Figure S6A). These results were further validated in the CGGA dataset (Figures S5B-H and S6B). Meanwhile, we also found that the risk signature has a high value in predicting prognosis of patients stratified by age or gender in both datasets (Figure S6C-D). Next, we performed univariate and multivariate Cox regression analyses with risk score and other well-known clinical factors. The results showed that the risk signature is an independent prognostic factor for glioma patients (Table 2 and Table S3). Finally, ROC curve analyses were performed to evaluate the predictive ability of the risk signature in predicting the survival rate. The results indicated that the risk score produces satisfactory AUC values for TCGA dataset (1-year: 89.7%, 2-year: 90.9%, 3-year: 91.1%, 4-year: 85.8%, and 5-year: 83.7%) (Figure 4(h)). Meanwhile, there were also high AUC values for the CGGA dataset (1-year: 78.2%, 2-year: 85.9%, 3-year: 86.9%, 4-year: 88.3%, and 5-year: 88.5%) (Figure 4(i)). Collectively, our risk signature harbors a robust prognostic value for glioma patients.

### 3.4. An Independent Prediction Model Based on the Risk Score, Age, Grade, and Status of 1p19q Codeletion

**Figure 5:**
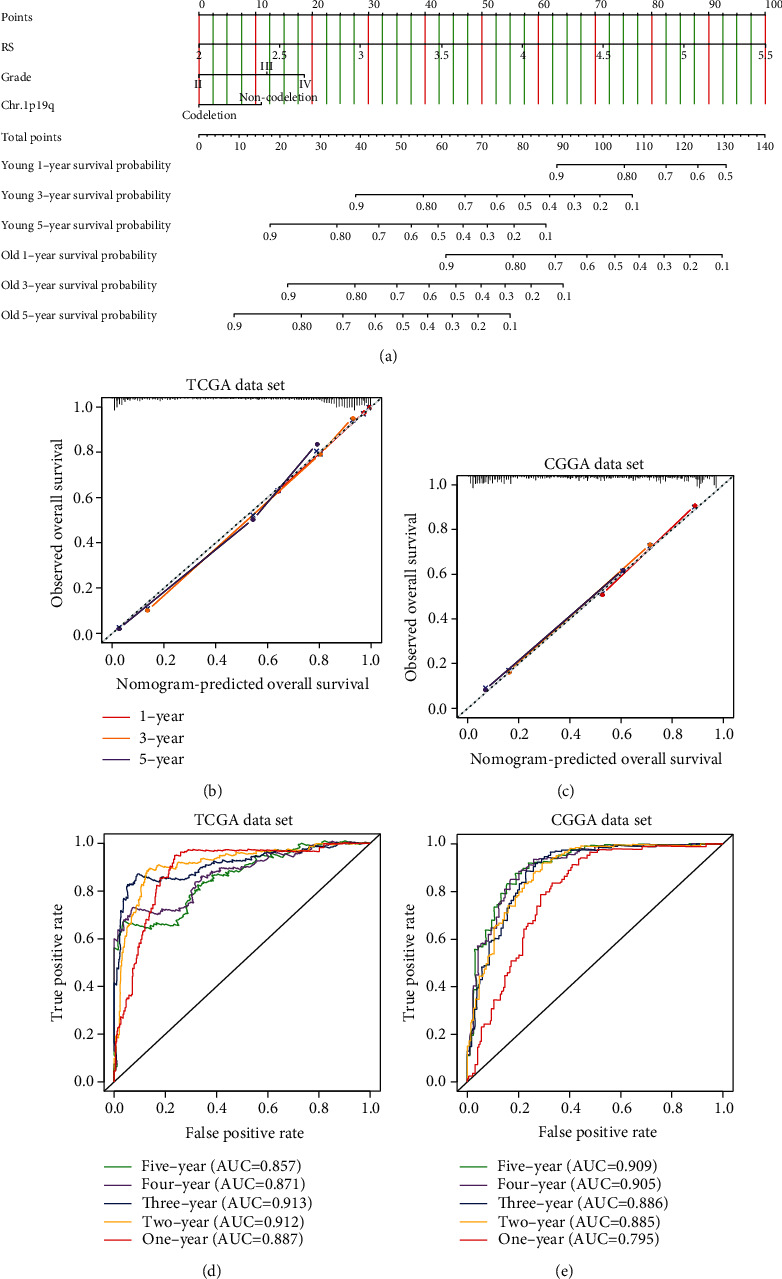
An independent prediction model for OS of glioma patients. (a) The nomogram predicting 1-year, 3-year, and 5-year OS for glioma patients in TCGA dataset. (b, c) Calibration plots predicting robustness of the nomogram at 1 year, 3 years, and 5 years in TCGA and CGGA datasets. (d, e) Time-dependent ROC curve analyses showing the predictive value of the score calculated by nomogram model for 1-year, 2-year, 3-year, 4-year, and 5-year OS in TCGA and CGGA datasets.

To explore the possibility to clinically apply our findings, we integrated the risk score and individualized clinicopathological parameters of glioma patients using the nomogram model. The C-indexes were 0.792 and 0.764 in TCGA and CGGA datasets, respectively, highlighting the satisfactory performance of this model (Figure 5(a)). Additionally, the calibration plots were constructed, and the results further validated the consistency of the model with patients' OS and in both datasets (Figures 5(b) and 5(c)). Next, we calculated the score of the nomogram model and further performed ROC curve analyses based on this score. The results showed that the integrated clinical model has significantly improved AUC values for TCGA dataset (1-year: 88.7%, 2-year: 91.2%, 3-year: 91.3%, 4-year: 87.1%, and 5-year: 85.7%) (Figure 5(d)) and the CGGA dataset (1-year: 79.5%, 2-year: 88.5%, 3-year: 88.6%, 4-year: 90.5%, and 5-year: 90.9%) (Figure 5(e)). These results indicate that our risk signature has high clinical application value.

### 3.5. Functional Annotation of the Risk Model

**Figure 6:**
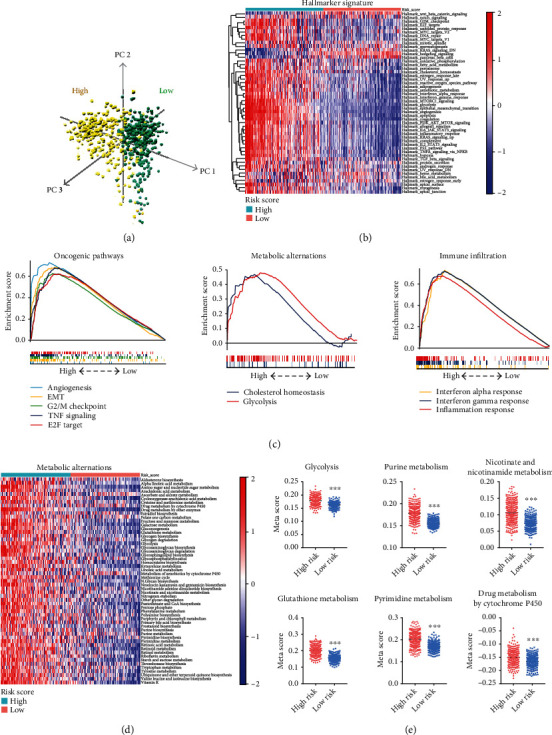
Functional analyses of the ECMRG signature in TCGA dataset. (a) PCA of differential gene expression profiles between the high-risk and low-risk groups. (b) Heat map plotting the metascore from GSVA analysis. GSVA analysis was performed to assess the pathway enrichment scores in each glioma sample in TCGA dataset. (c) GSEA showing the enriched oncogenic pathways, metabolic alternations, and immune infiltration in the high-risk group. (d) Heat map showing the elevated metabolic pathways for gliomas in the high-risk group. (e) Dot plots comparing the metascore of metabolic pathways for glioma patients between the high-risk and low-risk groups. ^∗∗∗^*P* < 0.001.

In TCGA and CGGA datasets, a principal component analysis (PCA) was constructed, and the results showed distinct transcriptional signatures between the high-risk and low-risk groups (Figure 6(a) and Figure S7A). Gene set variation analysis (GSVA) was performed to explore pathway activations in the high- and low-risk groups. The obtained metascore from GSVA was presented using a heat map (Figure 6(b)). The results showed that the high-risk score is positively associated with multiple oncogenic pathways such as epithelial-mesenchymal transition (EMT), E2F target, G2/M checkpoint, TNF-NF*κ*B signaling, and angiogenesis. Interestingly, we also found that metabolism pathways (glycolysis, cholesterol homeostasis) and immune regulation (inflammation, interferon alpha, and interferon gamma responses) were enriched in the high-risk group. These findings were confirmed by GSEA (Figure 6(c)). Furthermore, the same analyses were performed using the CGGA dataset, and the results were consistent with the above findings (Figure S7B-C). These results show that the ECMRG signature is tightly correlated with oncogenic pathways and that its ECMRGs have a significant impact on the metabolic regulation and modification of TIME in gliomas.

### 3.6. The Risk Signature ECMRGs Play Critical Roles in the Regulation of Metabolic Changes in Gliomas

Given the above findings, the role of the risk signature ECMRGs in the regulation of glioma metabolic pathways was further explored in TCGA and CGGA datasets. The list of 114 metabolic pathways was obtained from a previous study [26], and we calculated the metascore of each sample in different metabolic pathways using GSVA. Next, differential analyses identified 55 metabolic pathways that were enriched in the high-risk group and in both datasets (Figure 6(d) and Figure S7D). We found that multiple glioma-related metabolic pathways were enriched in the high-risk group, including glycolysis [32], nicotinate and nicotinamide metabolism [33], purine metabolism [34], pyrimidine metabolism [35], glutathione metabolism [36], and drug metabolism [37] (Figure 6(e) and Figure S7E). In conclusion, these findings show that the signature ECMRGs play crucial roles in the regulation of glioma metabolic pathways.

### 3.7. High-Risk Score Is Strongly Associated with Immune Suppression in Gliomas

**Figure 7:**
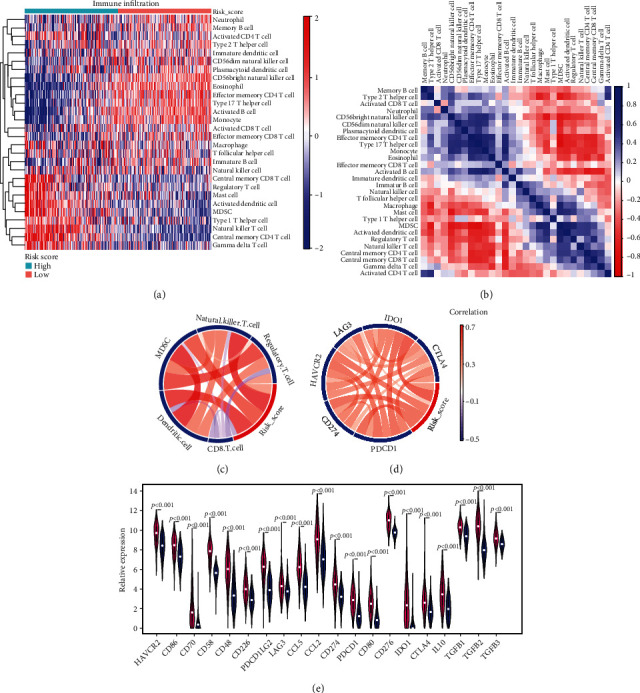
High-risk score is strongly associated with immune suppression in gliomas. (a) Heat map showing the infiltration of different immune cells in gliomas. ssGSEA was performed to assess the infiltration of each immune cell population in TCGA dataset. ssGSEA score was used for the heat map. (b) The correlation analysis between the immune infiltration and the risk signature in TCGA dataset. (c, d) CIRCOS plots showing the correlation between the infiltration of different immune cell populations (c)/immune checkpoint markers (d) and the risk signature. (e) Violin plot comparing the expression of immunosuppressive biomarkers between the high-risk and low-risk groups.

Due to the close relationship between ECM and multiple immune pathways in gliomas, we investigated immune infiltration in the high- and low-risk groups to characterize the immunologic landscapes. The abundance of 28 immune-related cell types was calculated using the ssGSEA algorithm and visualized in a heat map. The results suggested that among the 28 cell subpopulations, a high-risk score is positively correlated with regulatory T cells (Tregs), activated dendritic cells, myeloid-derived suppressor cells (MDSCs), and natural killer T cells (Figure 7(a)); however, the infiltration of activated CD8 T cells was negatively associated with the risk score. To confirm these findings, correlation analyses were performed which showed similar results (Figures 7(b) and 7(c)). Previous studies have well documented the immunosuppressive role of MDSCs and Tregs ^[31]^. Thus, the higher infiltration of MDSCs and Tregs suggested that a high-risk score is potentially linked with immune suppression in gliomas. To test our hypothesis, we compared the transcriptional expression of immune suppressive biomarkers between the high-risk group and the low-risk group, including immune checkpoint markers and secreted immune inhibitory factors. The results of correlation analysis indicated that the immune checkpoint markers, CD274 (PD-L1), PDCD1 (PD-1), CTLA-4, LAG3, HAVCR2 (TIM3), and IDO1, are positively associated with a high-risk score (Figure 7(d)). Consistently, a differential expression analysis showed that the selected inhibitory biomarkers have higher expression levels in the high-risk group (Figure 7(e)). The same analyses were conducted in the CGGA dataset, which yielded similar results (Figure S8). These results indicate that the signature ECMRGs play crucial roles in the regulation of tumors' immune status.

### 3.8. High-Risk Score Is Closely Linked with a Therapy-Resistant Phenotype in Gliomas

**Figure 8:**
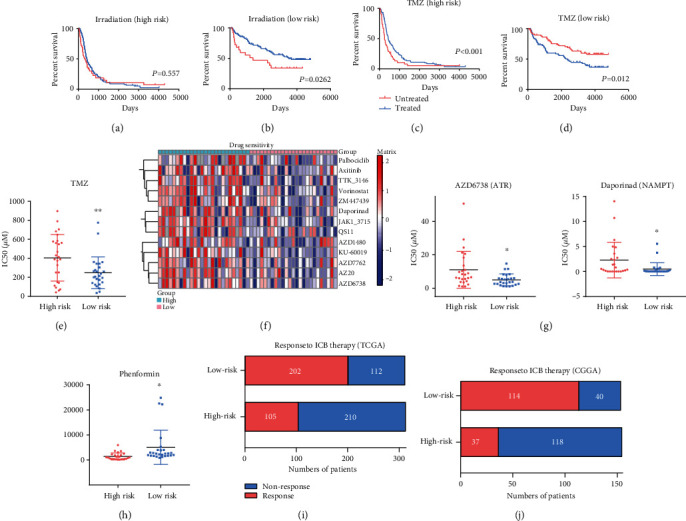
High-risk score is closely linked with a therapy-resistance phenotype in gliomas. (a, b) K-M curve analyses for glioma patients in the high-risk group (a)/low-risk (b) group treated with/without irradiation. (c, d) K-M curve analyses for glioma patients in the high-risk group (c)/low-risk (d) group treated with/without TMZ. (e) Dot plot comparing the IC50 of TMZ for glioma cell lines in the GDSC dataset between the high-risk and low-risk groups. (f) Heat map showing the response of glioma cell lines to multiple drugs in the GDSC dataset between the high-risk and low-risk groups. (g, h) Dot plots comparing the IC50 of AZD6738 (ATR kinase inhibitor) and daporinad (NAMPT inhibitor) (g) and phenformin (glycolysis and OXPHOS inhibitors) (h) for glioma cell lines in the GDSC dataset between the high-risk and low-risk groups. (i, j) Stacked bar charts comparing the response to ICB therapy between the high-risk and low-risk groups in TCGA (i) and CGGA (j) datasets. ^∗^*P* < 0.05,  ^∗∗^*P* < 0.01.

To further explore the clinical impact of the risk signature, we explored whether the ECMRG signature was linked with therapeutic response. Currently, irradiation and chemotherapy are the two first-line treatments after surgical resection. Thus, glioma patients were subgrouped based on their risk score, and K-M curve analyses were preformed to compare the OS of irradiation or temozolomide- (TMZ-) treated and untreated patients. The results showed that there is no statistical difference between irradiation-treated and untreated groups in patients with a high-risk score. However, the treated group had significantly longer OS compared with that in the untreated group in patients with a low-risk score (Figures 8(a) and 8(b)). For TMZ chemotherapy, the treatment improved the prognosis of both high-risk and low-risk score patients (Figures 8(c) and 8(d)). The results of survival analyses showed that a high-risk score is a strong indicator for radioresistance. However, these results were inconclusive for the TMZ treatment. Thus, we further analyzed the Genomics of Drug Sensitivity in Cancer (GDSC) dataset and explored the association between the risk signature and drug sensitivity. The LGG and GBM cell lines (52 in total) in the GDSC dataset were divided into the high-risk or low-risk groups based on the online available transcriptional data. The dot plot indicated that cell lines with a high-risk score have higher TMZ IC50 (Figure 8(e)), indicating the existence of a TMZ-resistant phenotype. To obtain a general understanding of the link between the risk signature and drug response, we analyzed the available drugs that were tested in the GDSC dataset. We found that the high-risk signature is strongly associated with resistance to multiple drugs, involving DNA repair, metabolic pathways, and cell cycle oncogenic kinases (Figure 8(f)). For example, dot plots showed that the IC50 of the ataxia telangiectasia and Rad3-related (ATR) kinase inhibitor (AZD6738) [38] and nicotinamide phosphoribosyltransferase (NAMPT) inhibitor (daporinad) [39] were significantly higher in the high-risk group (Figure 8(g)). We next sought to identify effective drugs that target the high-risk group. To our surprise, we found that among the screening inhibitors, the high-risk cell lines are only sensitive to phenformin, an inhibitor of glycolysis and oxidative phosphorylation (OXPHOS) [32] (Figure 8(h)). In addition, we further explored the response of our risk signature to ICB therapy using the TIDE algorithm. Similarly, the results showed that the high-risk group is an ICB therapy-resistant phenotype in both TCGA and CGGA datasets (Figures 8(i) and 8(j)). Together, these results indicate that the ECMRG signature may be a potential model for developing novel treatment strategies.

### 3.9. Functional Verification of BMP1 Oncogenic Role in Gliomas

**Figure 9:**
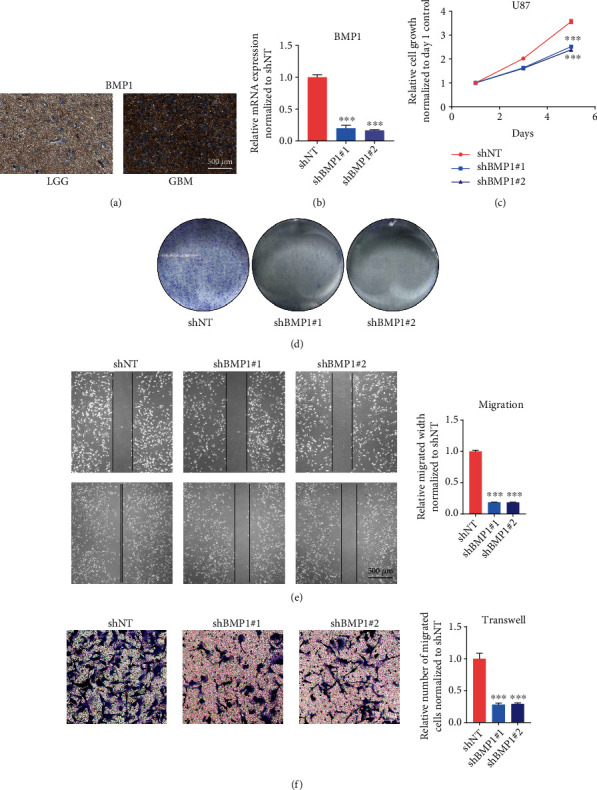
Functional verification of oncogenic role of BMP1 in gliomas. (a) The representative staining images for BMP1 in the Human Protein Atlas dataset. (b) qRT-PCR analysis detecting mRNA expression of BMP1 in U87 cells treated with lentiviral shBMP1#1, shBMP1#2, and shNT. (c) Cell viability assay detecting proliferative ability of U87 cells treated with lentiviral shBMP1#1, shBMP1#2, and shNT. (d) Colony formation assay detecting self-renewal ability of U87 cells treated with lentiviral shBMP1#1, shBMP1#2, and shNT. (e) The wound healing assay determining the migratory ability of U87 cells treated with lentiviral shBMP1#1, shBMP1#2, and shNT. (f) Transwell migration assay assessing the invasion ability of U87 cells treated with lentiviral shBMP1#1, shBMP1#2, and shNT. ^∗∗∗^*P* < 0.001.

The above results showed that BMP1 is elevated in gliomas. However, the expression and oncogenic role of BMP1 in gliomas have been rarely studied. Thus, we determined the protein expression level of BMP1 in the Human Protein Atlas (THPA) (https://www.proteinatlas.org/). As expected, the result showed that BMP1 is highly expressed in glioma samples. Moreover, there was a positive correlation between BMP1 protein expression and higher pathological grades (Figure 9(a)). For further verification, shRNAs targeting BMP1 (shBMP1 #1 and shBMP1 #2) were introduced into U87 glioma cells. qRT-PCR analysis was used to assess the efficacy of the BMP1 silencing. The results showed that the BMP1 mRNA expression is reduced in the shBMP1 group (Figure 9(b)). To further explore the oncogenic role of BMP1, cell viability assays were performed, and we observed that BMP1 silencing markedly decreased the growth of U87 cells (Figure 9(c)). In addition, colony formation assays were carried out, and the results indicated that the self-renewal ability of U87 cells that were transduced with shBMP1 is significantly attenuated (Figure 9(d)). Furthermore, wound healing assays and transwell migration assays were used to assess the oncogenic role of BMP1. The results showed that both the closure time and the number of invasive cells are significantly reduced in BMP1-knockdown U87 cells (Figures 9(e) and 9(f)). Taken together, these results suggest that BMP1 is functionally required for multiple malignant characteristics in gliomas.

## 4. Discussion

Several studies identified significant correlations between distinct molecular subtypes and clinical outcomes in glioma patients [4, 7, 8, 40, 41]. However, the targeted therapies for specific subtypes largely failed because of intratumoral heterogeneity [9]. Accumulating evidence indicated that the ECM has a critical impact on the invasive phenotype of multiple cancers, including gliomas [42]. Therefore, we aimed to construct an ECMRG signature that provides a better assessment model for clinical applications.

After comprehensive analyses, we constructed a risk model that contained 11 prognosis-related ECMRGs. Among the 11 genes, 10 biomarkers were highly expressed in the high-risk group and strongly associated with poor prognosis, including BMP1, CASP3, CD151, COL8A1, LOX, PLOD3, SDC1, SERPINH1, SPP1, and TIMP. Previous studies have reported the oncogenic role of these biomarkers in gliomas. For instance, recent evidence showed that elevated PLOD3 promotes gliomas' malignant characteristics and poor prognosis [43]. In addition, a study showed that LOX expression strongly correlates with the invasive features of malignant astrocytes [44]. Although several studies have reported the oncogenic role of BMP1 in other cancers, including non-small-cell lung cancer [45] and gastric cancer [46], the role of BMP1 in gliomas is unknown. Thus, we functionally verified the effects of BMP1 **o**n gliomas and showed that BMP1 is strongly associated with gliomas' invasive behaviors *in vitro*.

Accumulating evidence demonstrated a strong relationship between a high-risk score and multiple metabolic processes, including glycolysis [32], purine synthesis [34], pyrimidine synthesis [35], glutathione [36], nicotinate and nicotinamide metabolism [33], and drug metabolism by Cytochrome P450 [37]. The involvement of these processes in oncogenic progression has been well documented by previous studies. For instance, GBM cells have been shown to utilize glycolysis to maintain invasive growth [32]. In addition, a study demonstrated that purine synthesis can induce GBM radioresistance [34]. A recent study showed that glioblastoma stem cells (GSCs) utilize pyrimidine synthesis to maintain self-renewal, proliferation, and tumorigenesis [35]. Additionally, a study indicated that IDH1-mutated glioma has elevated demands for glutathione to sustain malignancies [36]. Collectively, the enrichment of these metabolic processes further highlights the clinical applicability of our risk model.

The immune-associated pathway enrichment in the high-risk group suggests a potential correlation between our risk model and the TIME in gliomas. To validate our hypothesis, we further explored the link between the risk signature and immune infiltration. The results uncovered positive correlations between the high-risk score and MDSCs and Tregs high infiltrations. MDSCs and Tregs have been shown to play critical roles in immunosuppression [47]. Moreover, the link between the risk model and the transcriptional expression of immunosuppressive biomarkers was also investigated. The high-risk score was found to be closely associated with elevated mRNA expression of immunosuppressive biomarkers, such as CD274 (PD-L1), LAG3, CTLA-4, and IDO1. Interestingly, previous studies reported that the engagement of PD-L1 with PD-1 can result in exhaustion of activated T cells [48, 49]. Indeed, a relatively lower level of CD8+ T cells was observed in the high-risk group. Clinically, a recent study demonstrated that the coblockade of TIM3 and PD1 improves anticancer T cell responses [50]. In addition, recent studies showed that anti-CTLA-4 and anti-PD1/PD-L1 combination therapy activates T cells during cancer treatment [51–54]. Collectively, a higher infiltration of immunosuppressive cells and elevated immunosuppressive biomarkers contribute to the immune escape in gliomas, by inactivating tumor killing cells such as CD8+ T cells. However, the detailed mechanism of the correlation between the risk model and key immune checkpoint markers is still unclear. A recent review demonstrated that PD-L1 regulatory mechanisms are affected by multiple levels, including its regulation at the DNA, RNA, and protein levels, and through extracellular secretion, indirect regulation by biomarkers, and potential drug intervention, while the regulation of CTLA-4 mainly depends on its cellular localization [55]. Thus, further functional studies are warranted to deepen our understanding of the underlying mechanisms.

Our study found that the risk signature is predictive of therapeutic response in clinical practice. The survival analysis demonstrated that patients with a high-risk score are more likely to benefit less from irradiation treatment. Consistently, a positive correlation between the risk signature and TMZ IC50 value was observed in the GDSC dataset, indicating that the high-risk group has an unsatisfactory response to TMZ. In addition, according to the GDSC dataset, we found that high-risk score glioma cell lines are resistant to multiple antitumoral inhibitors. Moreover, our study indicated that a high-risk score reflects a resistant phenotype to ICB therapy. Therefore, these findings suggest that our risk signature has practicability in evaluating therapeutic responses. However, detailed functional assays are necessary to verify our findings.

Recently, numerous glioma prognostic models have been reported. Thus, it is critical to assess the quality of the prediction models. A recent study performed a comprehensive analysis of the prediction models for gliomas. According to the study finding, the vital factors for assessing the model include performance estimation, validation, and event per variable (EPV) [56]. In our study, we explored the risk model from multiple perspectives and observed a much higher AUC value compared with that reported by previous studies [57–59]. Furthermore, we verified our risk model through external datasets, and EPV in our model was more than 10, highlighting the good quality of the ECMRG risk model. Nevertheless, our study has some limitations. Firstly, due to the unavailability of our own validation cohort, the study relied on data derived from a publicly available database. Secondly, immune infiltration was indirectly assessed via bioinformatics analyses. Thus, a functional verification using mouse models or single-cell RNA sequencing from glioma samples is necessary for further validation. Lastly, our study preliminarily explored the oncogenic role of BMP1 *in vitro*, and more in-depth studies are needed.

In conclusion, we identified and characterized a novel risk model that can be used as a valuable prediction tool in clinical assessment.

## Figures and Tables

**Table 1:** Characteristics of patients in cluster 1 and cluster 2 in TCGA dataset.

Characteristics	*N*	Cluster 1	Cluster 2	*P* value
Total cases	629	246	383	
Gender				0.182
Male	329	142	187	
Female	242	91	151	
Age (years)				<0.001
≤47	289	56	233	
>47	282	177	105	
Grade				<0.001
II	210	21	189	
III	228	67	161	
IV	144	142	2	
Subtype				<0.001
Classical	81	81	0	
Mesenchymal	90	85	5	
Proneural	223	26	197	
Neural	104	21	83	
IDH status				<0.001
Mutation	404	33	371	
Wild-type	218	209	9	
MGMT promoter				<0.001
Methylation	450	99	351	
Unmethylation	149	117	32	
1p19q				<0.001
Codel	157	3	154	
Non-codel	466	237	229	

**Table 2:** Univariate and multivariate analyses of risk score and clinical features in TCGA dataset.

Variables	Univariate analysis		Multivariate analysis	
	HR (95% CI)	*P* value	HR (95% CI)	*P* value
Risk score	5.697 (4.562-7.114)	<0.001	2.551 (1.574-4.135)	<0.001
Age	1.071 (1.059-1.083)	<0.001	1.042 (1.028-1.056)	<0.001
Gender	0.887 (0.660-1.192)	0.427		
WHO grade				
III	3.055 (1.987-4.697)	<0.001	1.677 (1.058-2.657)	0.028
IV	21.520 (13.491-34.329)	<0.001	2.326 (1.238-4.368)	0.009
IDH status	0.095 (0.069-0.131)	<0.001	0.911 (0.455-1.822)	0.791
MGMT status	0.317 (0.234-0.427)	<0.001	0.827 (0.582-1.176)	0.291
1p19q status	0.237 (0.147-0.382)	<0.001	0.600 (0.342-1.053)	0.075

## Data Availability

All data used in the present study can be downloaded from TCGA dataset (https://xenabrowser.net/datapages/) and the CGGA dataset (http://www.cgga.org.cn).

## Supplementary Materials

Supplementary Materials Table S1: characteristics of patients in cluster 1 and cluster 2 in the CGGA dataset. Table S2: LASSO regression coefficients of 11 survival-associated ECMRGs in gliomas. Table S3: univariate and multivariate analyses of risk score and clinical features in the CGGA dataset. Figure S1: consensus clustering for the ECMRGs in glioma patients in the CGGA dataset. (A) Consensus clustering CDF for *k* = 2 to *k* = 10. (B) Relative change in area under CDF curve for *k* = 2 to *k* = 10. (C) Consensus clustering matrix of 309 samples from the CGGA dataset for *k* = 2. (D) Heat map of 2 clusters constructed by the top 50 differential expression genes. (E) K-M survival analysis of patients from 2 clusters classified by consensus clustering. Figure S2: identification of the ECMRG signature in the CGGA dataset. (A) LASSO coefficient profiles of the most powerful prognostic genes. (B) Heat map showing the expression profiles of 266 ECMRGs and corresponding clinical characteristics in the CGGA dataset. (C-I) Dot plots comparing the risk score for glioma patients stratified by WHO grade, IDH mutation status, 1p/19q codeletion status, MGMTp methylation status, age, gender, or cluster groups of gliomas. (J) ROC curve analyzing the predictive role of the risk signature for cluster groups. ^∗∗∗^*P* < 0.001; ns: not significant. Figure S3: the prognostic value of 11 ECMRGs in TCGA dataset. K-M survival analyses comparing OS based on the median expression of 11 genes between the high-expression and low-expression groups in TCGA dataset. Figure S4: the prognostic value of 11 ECMRGs in the CGGA dataset. K-M survival analyses comparing OS based on the median expression of 11 genes between the high-expression and low-expression groups in the CGGA dataset. Figure S5: the prognostic value of the risk signature in the CGGA dataset. (A) Distribution of the risk score, survival status, and mRNA expression level of 11 genes in the risk signature. (B-H) K-M survival analyses comparing OS for glioma patients stratified by WHO grade, IDH mutation status, and 1p/19q codeletion status in the high-risk and low-risk groups. Figure S6: the prognostic value of the risk model for the patients stratified by MGMTp methylation status, age, and gender; K-M survival analyses comparing OS for glioma patients with high-risk and low-risk scores stratified by MGMTp methylation status, age, or gender in TCGA dataset (A, C) and CGGA dataset (B, D), respectively. Figure S7: functional analyses of the ECMRG signature in the CGGA dataset. (A) PCA of differential gene expression profiles between the high-risk and low-risk groups. (B) Heat map plotting the metascore from GSVA analysis. GSVA analysis was performed to assess the pathway enrichment scores in each glioma sample in the CGGA dataset. (C) GSEA showing the enriched oncogenic pathways, metabolic regulations, and immune infiltration in the high-risk group. (D) Heat map showing the critical metabolic pathways for gliomas in TCGA dataset. (E) Dot plot comparing the metascore of metabolic pathways for glioma patients between the high-risk and low-risk groups. ^∗∗∗^*P* < 0.001. Figure S8: high-risk score is strongly associated with immune suppression in gliomas. (A) Heat map showing the infiltration of different immune cells in gliomas. ssGSEA was performed to assess the infiltration of each immune cell population in the CGGA dataset. ssGSEA score was used for the heat map. (B) The correlation analysis between the immune infiltration and the risk signature in the CGGA dataset. (C, D) CIRCOS plots showing the correlation between the infiltration of different immune cell populations (C)/immune checkpoint markers (D) and the risk signature. (E) Violin plot comparing the expression of immunosuppressive biomarkers between the high-expression and low-risk groups.
