# Supplementary material for: A Novel Risk Score Model Based on Eleven Extracellular Matrix-Related Genes for Predicting Overall Survival of Glioma Patients
Source: J Oncol. 2022 Apr 29;2022:4966820. doi: 10.1155/2022/4966820 (PMC9076298; doi:10.1155/2022/4966820)
Supplement: Supplementary Materials — Table S1: characteristics of patients in cluster 1 and cluster 2 in the CGGA dataset. Table S2: LASSO regression coefficients of 11 survival-associated ECMRGs in gliomas. Table S3: univariate and multivariate analyses of risk score and clinical features in the CGGA dataset. Figure S1: consensus clustering for the ECMRGs in glioma patients in the CGGA dataset. (A) Consensus clustering CDF for k = 2 to k = 10. (B) Relative change in area under CDF curve for k = 2 to k = 10. (C) Consensus clustering matrix of 309 samples from the CGGA dataset for k = 2. (D) Heat map of 2 clusters constructed by the top 50 differential expression genes. (E) K-M survival analysis of patients from 2 clusters classified by consensus clustering. Figure S2: identification of the ECMRG signature in the CGGA dataset. (A) LASSO coefficient profiles of the most powerful prognostic genes. (B) Heat map showing the expression profiles of 266 ECMRGs and corresponding clinical characteristics in the CGGA dataset. (C-I) Dot plots comparing the risk score for glioma patients stratified by WHO grade, IDH mutation status, 1p/19q codeletion status, MGMTp methylation status, age, gender, or cluster groups of gliomas. (J) ROC curve analyzing the predictive role of the risk signature for cluster groups. ∗∗∗P < 0.001; ns: not significant. Figure S3: the prognostic value of 11 ECMRGs in TCGA dataset. K-M survival analyses comparing OS based on the median expression of 11 genes between the high-expression and low-expression groups in TCGA dataset. Figure S4: the prognostic value of 11 ECMRGs in the CGGA dataset. K-M survival analyses comparing OS based on the median expression of 11 genes between the high-expression and low-expression groups in the CGGA dataset. Figure S5: the prognostic value of the risk signature in the CGGA dataset. (A) Distribution of the risk score, survival status, and mRNA expression level of 11 genes in the risk signature. (B-H) K-M survival analyses comparing OS for glioma patients str [file 4966820.f1.pdf]

Supplementary tables

Table S1. Characteristics of patients in cluster 1 and cluster 2 in the CGGA dataset

Characteristics	N	Cluster1	Cluster2	p value
**Total cases**	309	170	139	
**Gender**				0.213
Male	194	112	82	
Female	115	58	57	
**Age (years)**				<0.001
≤42	155	68	87	
＞42	154	102	52	
**Grade**				<0.001
II	97	13	84	
III	73	39	34	
IV	135	114	21	
**IDH Status**				<0.001
Mutation	165	46	119	
Wildtype	143	124	19	
**MGMT Promoter**				0.093
Methylation	151	78	73	
Unmethylation	140	86	54	
**1p19q**				<0.001
Codel	62	10	52	
Non-codel	239	155	84	

Table S2. LASSO regression coefficients of 11 survival-associated ECMRGs in gliomas

Gene	value
BMP1	0.1037
BMP2	-0.1159
CASP3	0.0890
CD151	0.0076
COL8A1	0.0067
LOX	0.0230
PLOD3	0.0196
SDC1	0.0362
SERPINH1	0.0075
SPP1	0.0450
TIMP1	0.1363

Table S3. Univariate and multivariate analyses of risk score and clinical features in the CGGA dataset

Variables	Univariate analysis		Multivariate analysis	
	HR (95% CI)	p value	HR (95% CI)	p value
**Risk score**	3.046(2.506-3.703)	<0.001	2.102(1.559-2.834)	<0.001
**Age**	1.033(1.019-1.046)	<0.001	1.012(0.999-1.024)	0.061
**Gender**	0.995(0.748-1.323)	0.973		
**WHO Grade**				
**III**	3.138(2.010-4.900)	<0.001	2.364(1.478-3.782)	<0.001
**IV**	8.096(5.377-12.191)	<0.001	3.874(2.401-6.251)	<0.001
**IDH mutation**	0.305(0.261-0.469)	<0.001	1.907(1.256-2.897)	0.002
**MGMT status**	0.885(0.669-1.169)	0.389		
**1p19q status**	0.183(0.112-0.299)	<0.001	0.324(0.189-0.554)	<0.001

Supplementary figures and figure legends

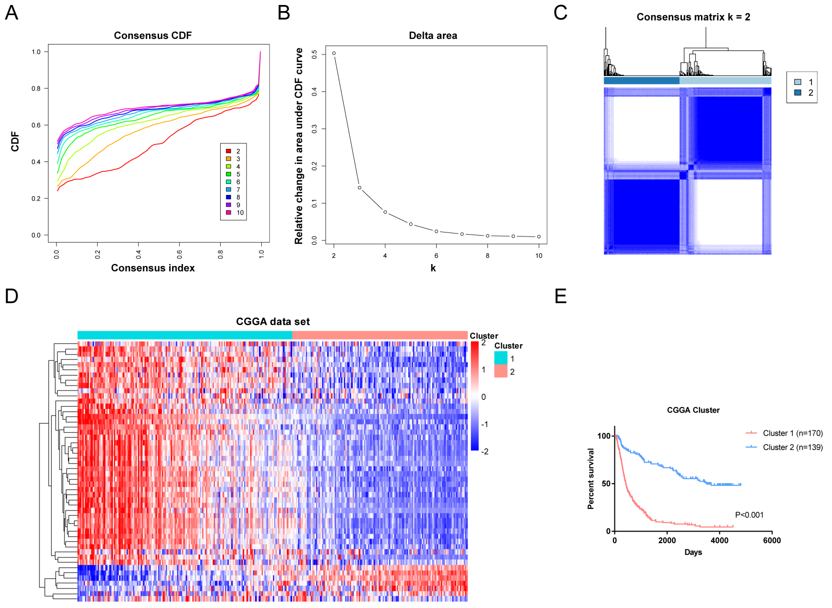

Figure S1. **Consensus clustering for the ECMRGs in glioma patients in the CGGA dataset**

(**A**) Consensus clustering CDF for k = 2 to k = 10. (**B**) Relative change in area under CDF curve for k = 2 to k = 10. (**C**) Consensus clustering matrix of 309 samples from the CGGA dataset for k = 2. (**D**) Heat map of 2 clusters constructed by the top 50 differential expression genes. (**E**) K-M survival analysis of patients from 2 clusters classified by consensus clustering.

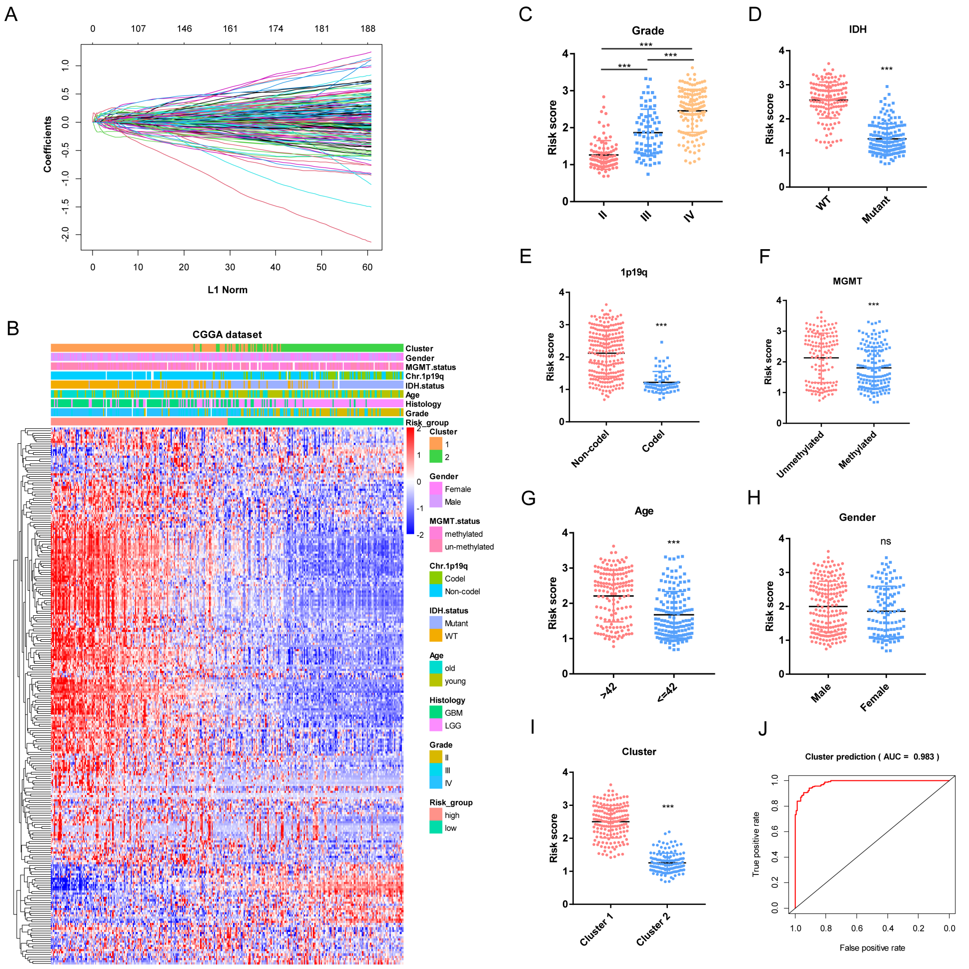

Figure S2. Identification of the ECMRG signature in the CGGA dataset

(**A**) LASSO coefficient profiles of the most powerful prognostic genes. (**B**) Heat map showing the expression profiles of 266 ECMRGs and corresponding clinical characteristics in the CGGA dataset. (**C-I**) Dot plots comparing the risk score for glioma patients stratified by WHO grade, IDH mutation status, 1p/19q codeletion status, MGMTp methylation status, age, gender or cluster groups of gliomas. (**J**) ROC curve analyzing the predictive role of the risk signature for cluster groups. *** $P < 0.001$, ns, not significant.

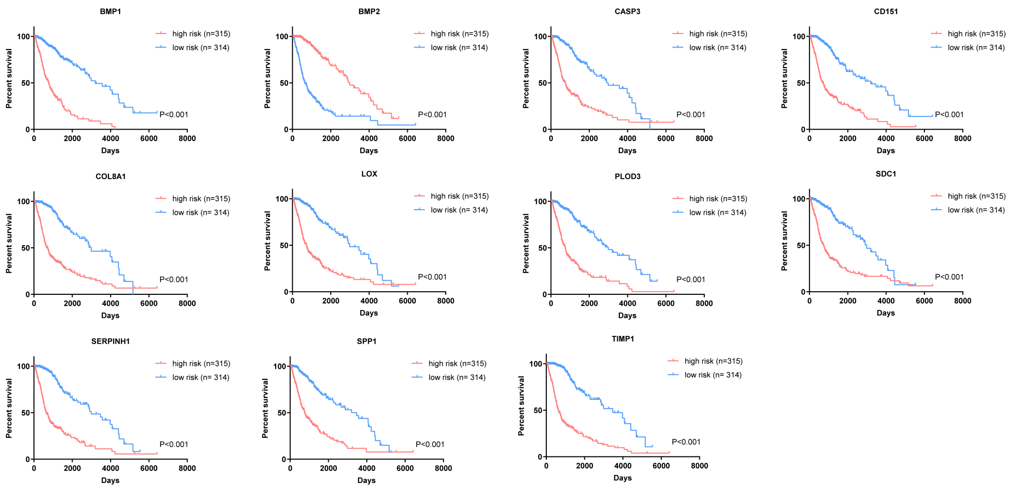

Figure S3. The prognostic value of 11 ECMRGs in TCGA dataset

K-M survival analyses comparing OS based on the median expression of 11 genes between the high-expression and low-expression groups in TCGA dataset.

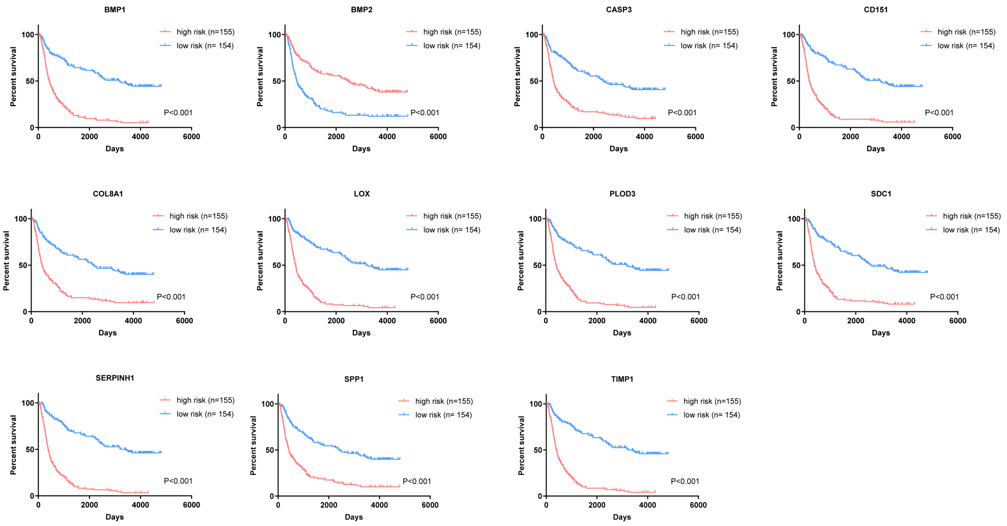

Figure S4. The prognostic value of 11 ECMRGs in the CGGA dataset

K-M survival analyses comparing OS based on the median expression of 11 genes between the high-expression and low-expression groups in the CGGA dataset.

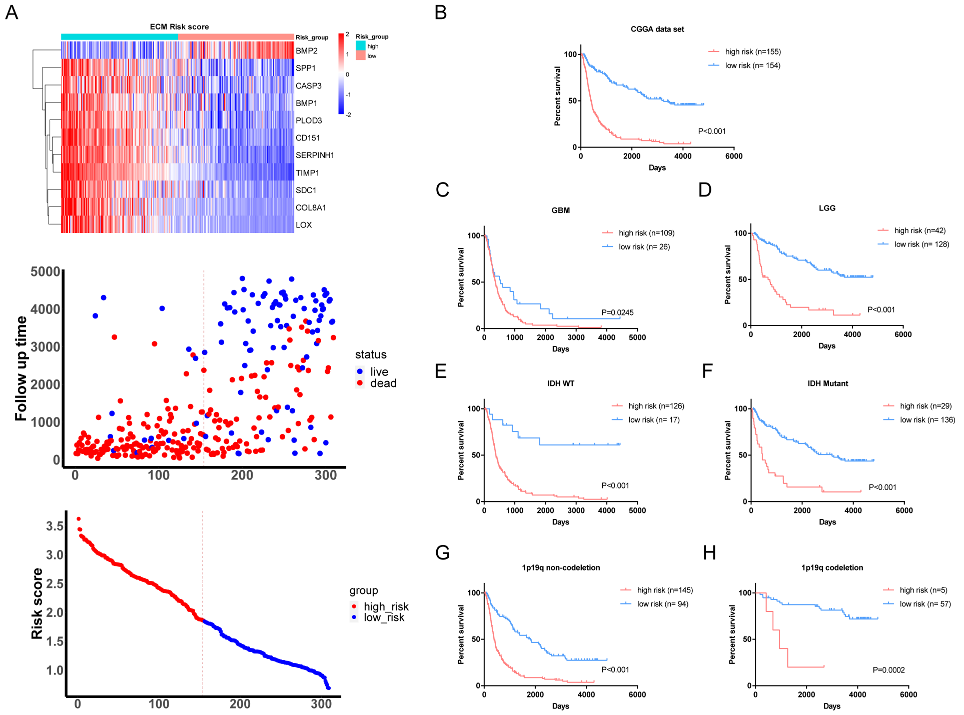

Figure S5. The prognostic value of the risk signature in the CGGA dataset

(**A**) Distribution of the risk score, survival status and mRNA expression level of 11 genes in the risk signature. (**B-H**) K-M survival analyses comparing OS for glioma patients stratified by WHO grade, IDH mutation status and 1p/19q codeletion status in the high-risk and low-risk groups.

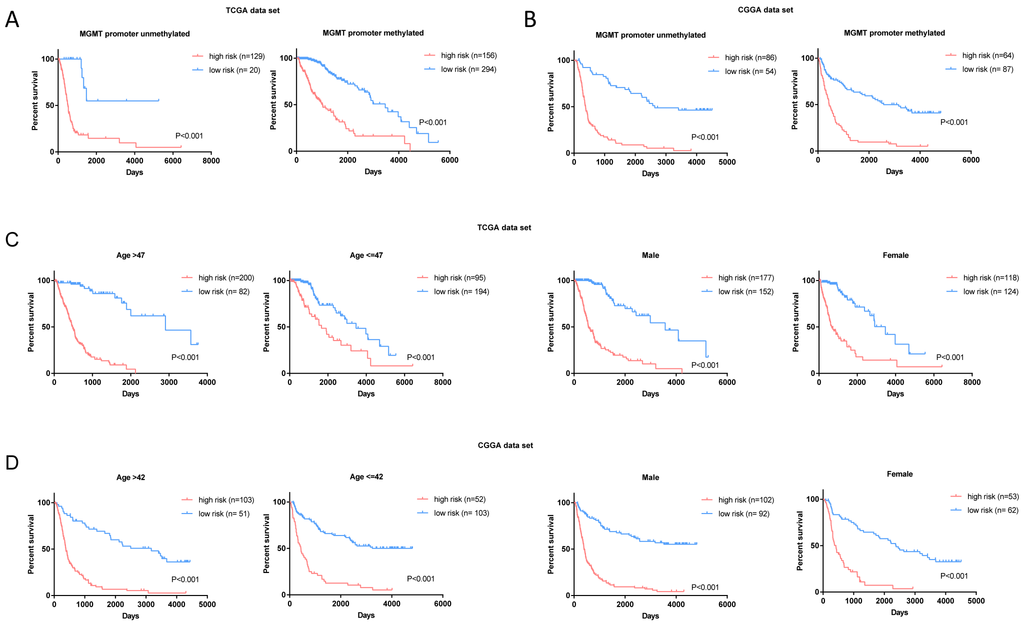

Figure S6. The prognostic value of the risk model for the patients stratified by MGMTp methylation status, age and gender

K-M survival analyses comparing OS for glioma patients with high-risk and low-risk score stratified by MGMTp methylation status, age or gender in TCGA dataset (**A, C**) and CGGA dataset (**B, D**), respectively.

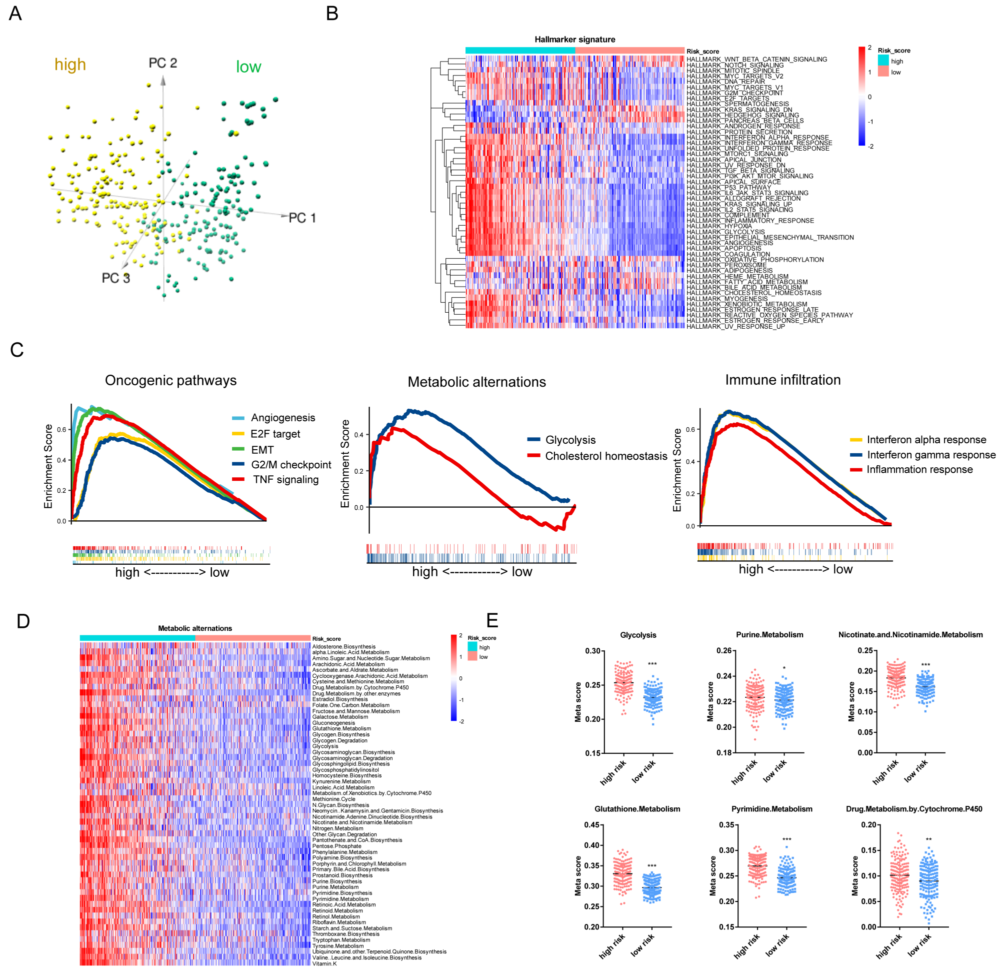

Figure S7. Functional analyses of the ECMRG signature in the CGGA dataset

(**A**) PCA of differential gene expression profiles between the high-risk and low-risk groups. (**B**) Heat map plotting the meta-score from GSVA analysis. GSVA analysis was performed to assess the pathway enrichment scores in each glioma sample in the CGGA dataset. (**C**) GSEA analyses showing the enriched oncogenic pathways, metabolic regulations and immune infiltration in the high-risk group. (**D**) Heat map showing the critical metabolic pathways for gliomas in TCGA dataset. (**E**) Dot plot comparing the meta-score of metabolic pathways for glioma patients between the high-risk and low-risk groups. *** P < 0.001.

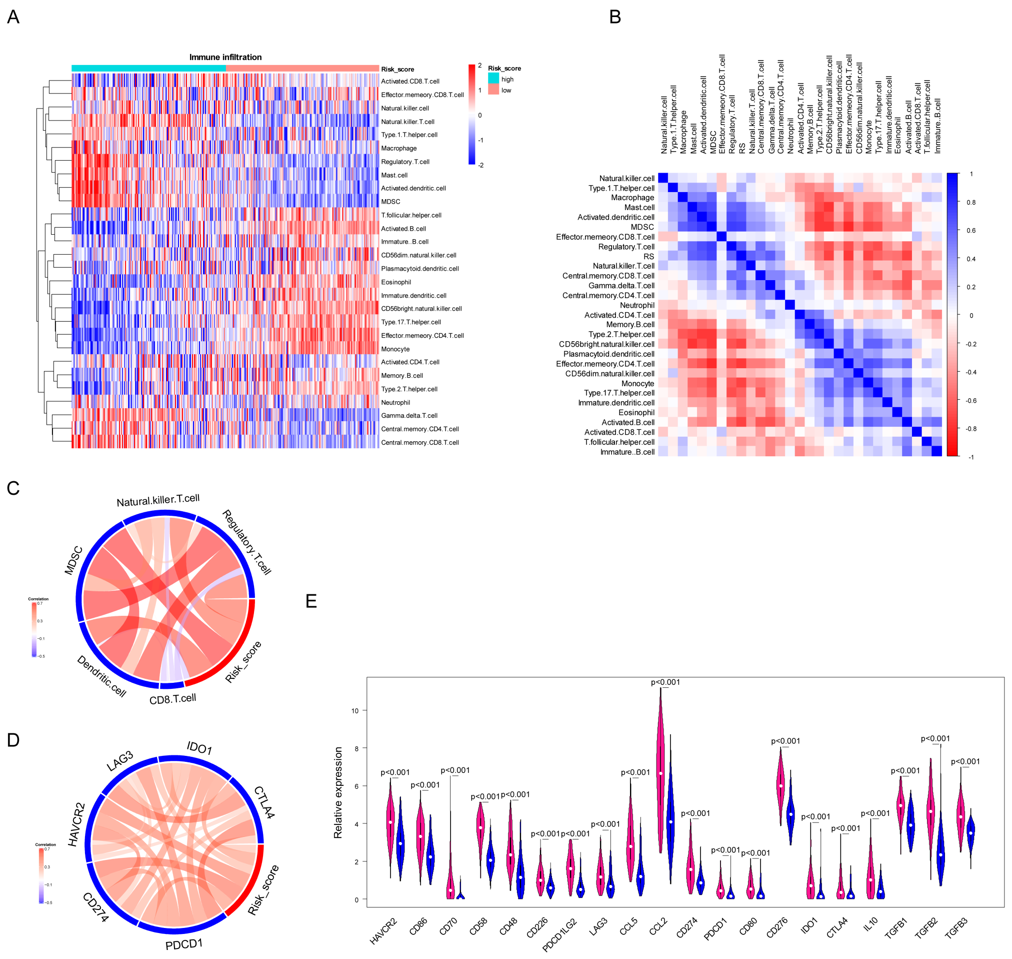

Figure S8. High-risk score is strongly associated with immune suppression in gliomas

(**A**) Heat map showing the infiltration of different immune cells in gliomas. ssGSEA analysis was performed to assess the infiltration of each immune cell population in the CGGA dataset. ssGSEA score was used for the heat map. (**B**) The correlation analysis between the immune infiltration and the risk signature in the CGGA dataset. (**C, D**) CIRCOS plots showing the correlation between the infiltration of different immune cell populations (**C**)/immune checkpoint markers (**D**) and the risk signature. (**E**) Violin plot comparing the expression of immunosuppressive biomarkers between the high-expression and low-risk groups.
